# Increased inflammation and oxidative stress caused by accumulated metal particle exposure among metro station staff

**DOI:** 10.1371/journal.pone.0337592

**Published:** 2025-12-10

**Authors:** Zukun Wang, Junjie Liu, Yijun Song, Mingyao Yao, Shihao Wen, Wenzhe Shang, Mingtong He, Yushuang Wang, Junjie He

**Affiliations:** 1 Tianjin Key Laboratory of Indoor Air Environmental Quality Control, School of Environmental Science and Engineering, Tianjin University, Tianjin, China; 2 Department of Intensive Care Medicine, State Key Laboratory of Experimental Hematology, National Clinical Research Center for Blood Diseases, Haihe Laboratory of Cell Ecosystem, Institute of Hematology and Blood Diseases Hospital, Chinese Academy of Medical Sciences and Peking Union Medical College, Tianjin, China; National Institutes of Health, University of the Philippines Manila / De La Salle University, PHILIPPINES

## Abstract

Metro is a significant part of world transport, delivering over 58 billion passengers annually. Train operation processes generated PM rich in heavy metals in the station, but the natural ventilation underground is poor leading to a high PM exposure indoors. Though PM pollution in metro stations was reported widely, there is limited evidence of the adverse health effect of metro station PM. This study collected urinary samples from 74 metro station staff from three different metro stations in Tianjin for 8-OHdG, IL-6, MDA, GSH and T-AOC tests using commercial kits. PM samples were collected for metal composition tests using ICP-MS and oxidative potential test using DTT method. The average PM_2.5_ concentration was 31.5 ± 12.4 μg/m^3^, meeting the Standard for indoor air quality in China. However, the indoor concentrations of Zn, Cu, Mn, and Fe were ten times higher than those in the ambient air, inferring a higher exposure level of metal particles. Urinary 8-OHdG, MDA, and IL-6 of senior employees (length of service in the metro station > 5 years) were significantly higher than new employees but not related to age, suggesting a significant influence on individual’s inflammation and oxidative stress led by PM exposure. Identified as characteristic elements in the metro stations, Mo and Ni, were also found significantly correlated with urinary IL-6 and GSH. The findings indicates that chronic metal PM exposure in the metro station may induce oxidative stress and inflammation. However, the accumulated PM_2.5_ concentration showed a poor relation with biomarkers except for IL-6. Accumulated oxidative potential of PM was significantly correlated with urinary IL-6, 8-OHdG, GSH, and T-AOC. This result suggested the accumulated oxidative potential of PM as a better evaluation method of health influence led by PM rather than mass concentration.

## 1. Introduction

Metro serves as a significant part of world transport. Metro system delivered over 58 billion passengers globally in 2019, among which over 30 billion passengers from the Asia-Pacific region [1]. Tokyo owned the world’s largest metro network, with annual ridership of four billion [1]. In China, the Shanghai and Beijing metro networks carried over four billion passengers annually [1]. Though frustrated by the COVID-19 pandemic, metro ridership has rebounded rapidly. After the pandemic restriction over on March 2023, monthly metro ridership in Tianjin increased to 50 million [2], marking an 84.8% rise over the same period last year [3]. As urbanization improves, metro system construction will proceed and serve more passengers.

Metro stations are typically situated underground, resulting in a limited natural ventilation effect indoors. With particulate matter (PM) generated by friction between train wheel and rail, then spread into the public area by piston wind, PM pollution became the main indoor air quality issue in the metro station. Indoor air quality in metro stations has drawn global concern in recent years, with attention focused on various countries, including China [4–6], Korea [7,8], Spain [9,10], Italy [11], Britain [12], USA [13]. According to a questionnaire survey in Beijing metro stations [14], nearly half of the passengers complained about the indoor air quality in the metro stations in winter. Yu et al. [15] demonstrated that the average PM1 exposure for a single metro trip in Shanghai would be 122 ± 77 μg/m^3^. Meanwhile, Train operation processes include the friction between wheel, brakes, and railway, generating particulate matter rich in metals such as Fe, Cr, Co, Ni, Mn, and Zn [16–18]. Studies found metal oxide particles in nano size [19,20]. Ultrafine particles have been shown to cause oxidative stress and inflammatory effects due to larger surface area [21,22]. Meanwhile, metal particles may also induce oxidative stress due to the participation of redox-active trace metals in free radical generation, such as Fe and Mn [23,24]. Targeting astrocytes, the oxidative stress and inflammation caused by inhaled metal particles may be related to neurodegeneration disease including Alzheimer’s disease [25,26]. Due to the difference on chemical composition between metro PM and ambient, the present recommended PM concentration limit for above-ground public building indoor environment may not be applicable for PM control in the metro station. It is necessary to consider the combined influence of chemical and physical characteristics when evaluating the adverse health effect of metro PM.

Studies were conducted to assess the influence of metro station PM on public health. It was suggested that the underground metal PM may cause adverse health effects in vivo studies [27]. An acute exposure experience resulted in a statistically significant increase in the level of fibrinogen and the count of regulatory T-cells expressing CD4/CD25/FOXP3 after volunteers were exposed in the metro station for two hours, indicating an escalation in inflammation [28]. An increased urinary DNA oxidative stress biomarker in underground workers was detected [12,13]. The primary pollutant in the metro station is particulate matter rich in metals. Meanwhile, studies found inhaled ultrafine manganese oxide particles in rats’ central nervous system [29]. Particulate matter would cause oxidative stress and thus, trigger inflammation and diseases [30–32]. Though toxicological researches had proved that PM in underground metro stations may be more toxic than that of ambient, a critical review [33] still found evidence proving the toxicity of metro station PM is limited, especially in vivo study that helps to understand mechanism and potential biomarker.

Thus, further research was required to determine the influence of metro PM on individual’s inflammation and oxidative stress biomarker. This study aimed to find the potential relation of health effect induced by accumulated PM exposure rich in metals and its potential evaluation method. The 8-OHdG (8-hydroxy-2’-deoxyguanosine) is considered a biomarker of oxidative DNA damage. Studies found high 8-OHdG correlated with cardiovascular disease and Alzheimer’s disease [34,35]. MDA (malondialdehyde) is the product of lipid peroxidation that would result in cellular damage, considered a bioindicator of cancer and pulmonary disease [36]. GSH (reduced glutathione) is the most abundant intracellular small thiol, participating in a variety of detoxification reactions [37]. GSH metabolism is linked with diseases such as cancer and neurodegeneration. IL-6 (interleukin-6) plays an irreplicable role in the infectious and inflammatory process in the human body, related to immune disease and neuroinflammation [38,39].

To explore the potential inflammation and oxidative stress induced by metal particulates, metro station staff, who were chronically and consistently exposed to metro station PM, were recruited from three different metro stations. Urine samples were collected and five oxidative stress and inflammation biomarkers, namely 8-OHdG, MDA, GSH, T-AOC, and IL-6, were tested. Indoor air quality investigation including temperature, humidity, CO_2_ concentration, PM_1_ and PM_2.5_ concentration in the metro station was conducted simultaneously. PM_1_ and PM_2.5_ samples were collected for element composition analysis and oxidative potential analysis using the dithiothreitol (DTT) method.

## 2. Methods

### 2.1. Location and test period

**Fig 1:**
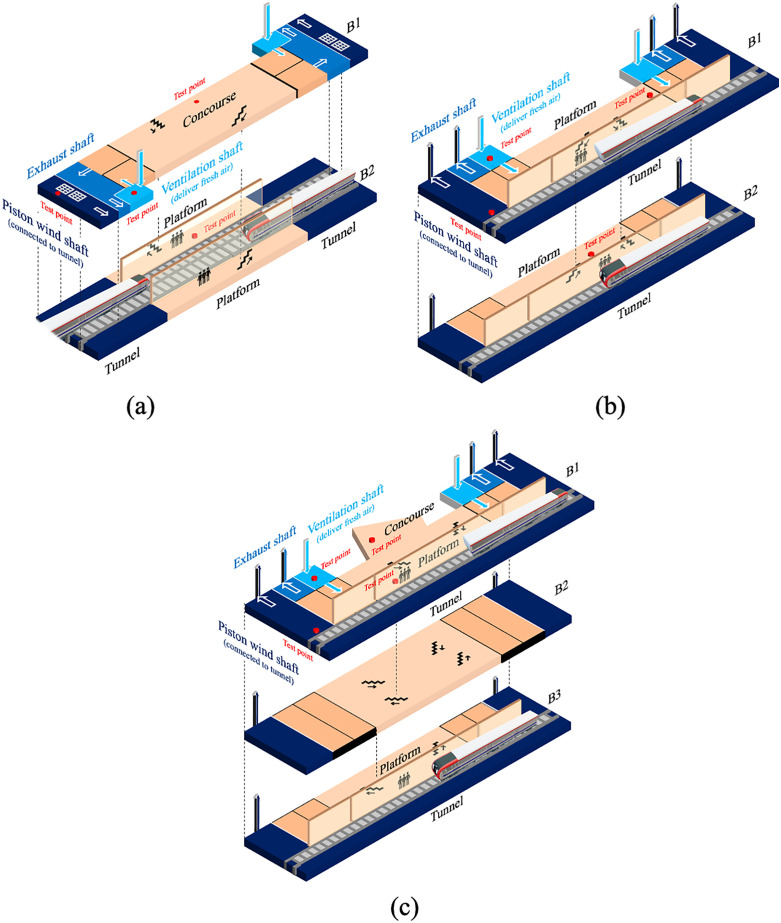
Sketch of Stations and test points. (a) Station A, (b) Station B, (c) Station C.

Measurement was conducted from June 21^st^ to July 17^th^, 2023, from 6:00–19:00 on workdays. Three metro stations, including Xinanlou Station (Station A), Jintanglu Station (Station B), and Chenglindao Station (Station C), serving the same metro line, located within a 5 km diameter circle, were selected. These metro stations had side platforms, with a single side train passing behind the full-height screen doors. Measurements and PM collection were conducted at the concourse, platform, ventilation shaft, and piston wind shaft. The sampling sites are marked with red dots in Fig 1. The ventilation shaft was considered ambient, as it delivered fresh air into the ventilation system. Located next to the train tunnel, the piston wind shaft exhaust piston wind driven by the train, representing air condition in the train tunnel.

### 2.2. Indoor air quality parameters

#### 2.2.1. Measurement of the environmental parameters.

The indoor air temperature, relative humidity, and carbon dioxide (CO_2_) concentration were recorded using a HOBO CO_2_ MX data logger (Onset, US) on the platforms and concourse every five minutes. The CO_2_ sensor was calibrated manually in the ventilation shaft (as ambient, 400 ppm). The hourly PM_2.5_ was monitored and recorded by DustTrak 8530 devices (TSI, US) on the platform, concourse, and piston wind shaft. Hourly ambient PM_2.5_ concentration was obtained from the Tianjin Ecology and Environment Bureau (https://sthj.tj.gov.cn/).

#### 2.2.2. PM chemical compositions analysis.

According to the HJ 657−2013 standard of China (Ambient Air and Stationary Source Emissions – Determination of Metals in Ambient Particulate Matter – Inductively Coupled Plasma/Mass Spectrometry (ICP‒MS)), PM_1_ and PM_2.5_ were gathered by TC-120F(S) PM samplers (SuYuan Environmental Protection Equipment Co. Ltd., Qingdao, Shandong, China) and XA-1000 PM samplers (QDXAO Environmental Technology Co. Ltd., Qingdao, Shandong, China), respectively, on 450 °C, 6h prebaked square glass fiber filters with pore sizes of 0.30 μm. Every PM sample was collected for 9h, with an airflow rate of 0.1 m^3^/min and 1.0 m^3^/min, respectively. Samples were sealed and stored at −18 °C before tests. A total of 72 samples were sent for element analysis using ICP-MS within one month.

Element analysis was conducted by ICP‒MS (Agilent ICP‒MS 7800). For preprocessing, the PM sample was cut into pieces using ceramic scissors and added to 10 mL of aqua regia for microwave digestion at 200 °C for 15 min. Cooling down to room temperature, the mixture was diluted using 10 mL pure water. Standing for 30 min, the liquid was filtered, then diluted into 50 mL. The sample supernatant after centrifugation was used for ICP‒MS analysis. A total of 24 elements were determined, including Al (Aluminium), P (Phosphorus), Cr (Chromium), Mn (Manganese), Fe (Iron), Co (Cobalt), Ni (Nickel), Cu (Copper), Zn (Zinc), Ga (Gallium), Rb (Rubidium), Sr (Strontium), Zr (Zirconium), Nb (Niobium), Mo (Molybdenum), Sn (Tin), Sb (Antimony), Ce (Cerium), Sm (Samarium), Gd (Gadolinium), Hf (Hafnium), Ta (Tantalum), W (Tungsten), Pb (Lead).

Enrichment factor (EF) was calculated to identify the characteristic element in the metro station as follows:


$$\begin{array}{c} EF_{element}=\frac{EC_{i}}{EC_{Al}}/\frac{EC_{crust\_i}}{EC_{crust\_Al}} \end{array}$$ (1)

In Equation (1), $EC_{i}$ and $EC_{Al}$ are the concentration of a specific element i and Al in samples. $EC_{crust\_element}\;$ and $EC_{crust\_Al}$ are the concentration of element i and Al in crust. An EF > 5 indicates a significant internal source element [40–42].

#### 2.2.3. Oxidative potential analysis.

The dithiothreitol (DTT) method was applied as a cell-free method for analyzing the oxidative potential (OP) of PM following steps described in previous studies [6,43]. In brief, a portion of a quartz filter was extracted in deionized water. The thiol groups were liberated using DTT (dithiothreitol) in a heated incubation. The reaction was then stopped with trichloroacetic acid. The liberated thiols were quantified using a colorimetric assay by reacting with DTNB (Ellman’s reagent) to produce a yellow-colored product (TNB). The intensity of this color, measured at 412 nm using a spectrometer, is proportional to the original thiol concentration. Details were supplied in Supplementary Material.

#### 2.2.4. Accumulated exposure assessment.

Accumulated PM_2.5_/PM_1_/element/oxidative potential exposure of a specific employee were calculated as follows:


$$\begin{array}{c} {AE}_{PM}=Y\cdot \text{3}\text{6}\text{5}d\cdot (aC_{p\_PM}+bC_{c\_PM}) \end{array}$$ (2)

$$\begin{array}{c} {ACE}_{i}=Y\cdot \text{3}\text{6}\text{5}d\cdot (a{EC}_{p\_i}+b{EC}_{c\_i}) \end{array}$$ (3)

$$\begin{array}{c} {AE}_{OP}=Y\cdot \text{3}\text{6}\text{5}d\cdot (a{OP}_{p\_PM}+b{OP}_{c\_PM}) \end{array}$$ (4)

In Equation (1), ${AE}_{PM}$ was the accumulated PM exposure for an employee; Y was his/her length of service in the metro station; $C_{p\_PM}$ and $C_{c\_PM}$ were the daily averaged PM concentration of platform and concourse, respectively; *a* and *b* were time-weighted fractions of the employee work at platform and concourse, respectively. In Equation (2), ${ACE}_{i}$ was the accumulated exposure of element *i* for an employee; ${EC}_{p\_i}$ and ${EC}_{c\_i}$ were the daily averaged element concentration on the platform and concourse, respectively. In Equation (3), ${AE}_{OP}$ was the accumulated oxidative potential exposure for an employee; ${OP}_{p\_PM}$ and ${OP}_{c\_PM}$ were the daily averaged oxidative potential of PM_2.5_ or PM_1_ on platform and concourse, respectively.

### 2.3. Urinary inflammation and oxidative stress biomarkers

The Tianjin Medical University General Hospital ethics committee approved this study (IRB2022-YX-256–01). A total of 74 volunteers working in metro Station A, B, and C were recruited, with basic information summarized in Table 1. Written informed consent was obtained from all participants after the experiments were described and clarified. Urine samples of volunteers were collected during work for biomarker analysis, including 8-hydroxy-2’-deoxyguanosine (8-OHdG), interleukin-6 (IL-6), malondialdehyde (MDA), reduced glutathione (GSH) and total antioxidant capacity (T-AOC). Informed consent was obtained from all participants after the experiments were described and clarified.

**Table 1:** Participants basic information.

Station	Station A	Station B	Station C	Total
Number of participants	20	36	18	74
Proportion of male	45%	50%	50%	49%
Proportion of smokers	20%	26%	17%	22%
Average age	29.5 ± 9.2	35.8 ± 12.7	35.7 ± 11.9	34.1 ± 11.8
Average service of year	4.0 ± 5.2	3.5 ± 3.6	5.8 ± 4.1	4.2 ± 4.2
Average BMI	26.6 ± 6.9	29.5 ± 13.7	26.4 ± 7.6	27.7 ± 10.4

The urinary levels of 8-OHdG and IL-6 were measured by using the commercially available ELISA kits (Shanghai Enzyme-linked Biotechnology, China), with supernatant after 3000 rpm centrifugation for 10 min. Urinary T-AOC was measured using commercial 2,2’-azino-bis(3-ethylbenzthiazoline-6-sulfonic acid) (ABTS) kits (Suzhou Comin Biotechnology, China), tested with supernatant after 5000 rpm centrifugation for 10 min. MDA was measured using the thiobarbituric acid (TBA) method (Shanghai Enzyme-linked Biotechnology, China). GSH was measured using the 5,5’-Dithiobis-(2-nitrobenzoic acid) (DTNB) method with commercial kits (Shanghai Enzyme-linked Biotechnology, China).

### 2.4. Statistical analysis

Statistical analysis was conducted using the Python statsmodels library [44]. To assess normality before comparison, the Shapiro-Wilk normality test was conducted. After normalizing the elemental concentration with z-score, linear mixed effect model was used to test the correlation of oxidative potential and element mass involved in DTT consumption in Section 3.2. Group comparison was conducted by T-test to evaluate the differences in the biomarker of metro station staff between different gender, smoker and non-smoker, obesity (BMI > 25) and non-obesity group, new (length of service in metro station < 5 years) and senior employee group in Section 3.3. The result was verified by linear mixed effect models. Linear mixed effects models were used for correlation analysis among biomarkers and accumulated PM/ metal/ oxidative potential exposure, balancing background factors, i.e., gender, smoking habit, BMI, length of service reported in Section 3.4. The confidence level is p value < 0.05 for all statistical analysis. Adjusted effect estimates were reported in this article.

## 3. Results

### 3.1. Indoor air quality and oxidative potential

The mean ± standard deviation (SD) of the ambient air temperature and relative humidity were 31.0 ± 4.0 °C and 50.8 ± 19.1%, respectively. Temperature, humidity and CO_2_ concentration during measurement were summarized in Table 2. The air temperature and relative humidity remained stable in the public areas of metro stations. The ventilation rate was enough to maintain an indoor CO_2_ concentration under 1000 ppm. No significant difference found among stations (p > 0.05).

**Table 2:** Indoor air environment during test.

Platform	Station A	Station B	Station C
Temperature, °C	22.9 ± 0.9	23.9 ± 0.7	23.7 ± 0.7
Humidity, %	64.6 ± 5.7	73.0 ± 5.4	65.3 ± 1.9
CO_2_, ppm	457.6 ± 51.7	441.0 ± 52.2	487.4 ± 38.1
Concourse	Station A	Station B	Station C
Temperature, °C	25.3 ± 1.2	26.0 ± 1.5	24.6 ± 0.5
Humidity, %	59.3 ± 6.3	74.7 ± 6.9	75.2 ± 4.4
CO_2_, ppm	478.2 ± 53.1	481.5 ± 63.8	505.8 ± 59.0

**Fig 2:**
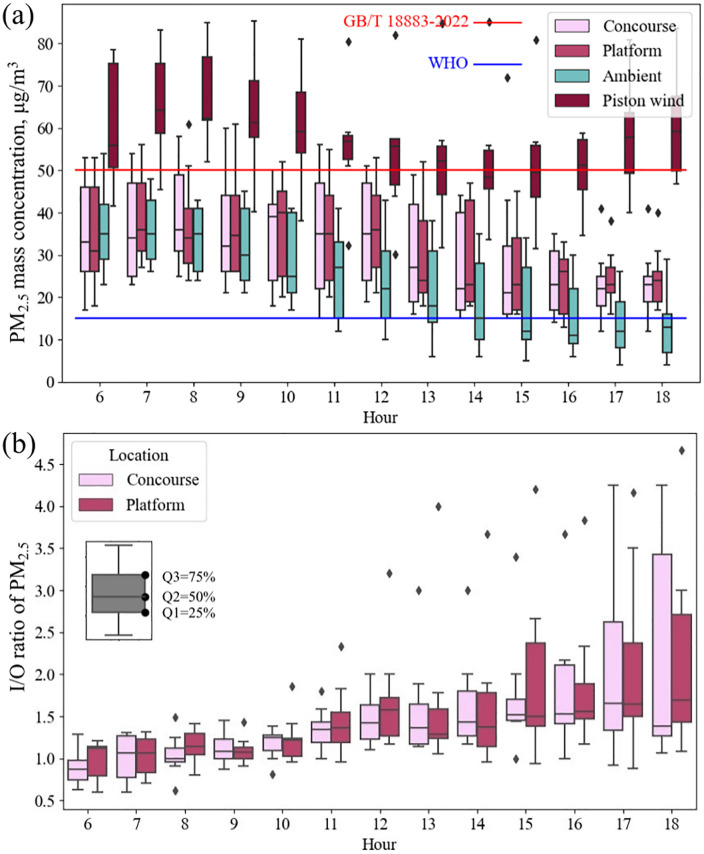
Hourly-averaged PM_2.5_ concentration and indoor/outdoor ratio in metro stations. (a) PM_2.5_ concentration; (b) Hourly-averaged indoor/outdoor (I/O) ratio of PM_2.5_.

The distribution of hourly PM_2.5_ was illustrated in Fig 2(a). The total average PM_2.5_ concentration was 31.5 ± 12.4 μg/m^3^ during the test duration. The indoor PM_2.5_ met the standard for indoor air quality (GB/T 18883–2022) with a recommended PM_2.5_ limit of 50 μg/m^3^ for more than 90% the test period. However the indoor PM_2.5_ concentration was still one time higher than the recommendation of World Health Organization (WHO), 15 μg/m^3^ [45]. The PM_2.5_ in the piston wind shaft peaked during the morning (7:00–10:00) with the maximum mass concentration reaching 89 μg/m^3^ and evening rush hour (16:00–19:00), when the train arrival interval was shorted from 6-8 min to 4.5 min. From the beginning of daily operation (6:00), the hourly-averaged PM_2.5_ on the platform, the concourse gradually decreased along time, as well as the ambient. However, the hourly-averaged I/O ratio in the metro stations went upward during the measured period, as shown in Fig 2(b). I/O ratios were above 1 most time, suggesting an internal PM_2.5_ source. Based on the PM_2.5_ concentration, piston wind in the train tunnel was inferred to be the source, spreading PM to the platform and concourse.

The oxidative potential of PM_2.5_ per unit volume of air in the metro tunnel was the highest among the test locations. The average oxidative potential of PM_1_ and PM_2.5_ per unit volume of air in metro tunnels was 10.2 nM/min·m^3^ and 10.9 nM/min·m^3^, respectively. Influenced by concentration, the oxidative potential per unit volume of air on the platforms was higher than that in the ambient and concourse. The average oxidative potentials of PM_1_ and PM_2.5_ per unit volume of air in the concourse were 1.7 nM/min·m^3^ and 2.1 nM/min·m^3^, while those in the ambient were 1.9 nM/min·m^3^ and 1.6 nM/min·m^3^, respectively. The average oxidative potential of PM_1_ and PM_2.5_ per unit volume of air at the station is 3.6 nM/min·m^3^ and 2.4 nM/min·m^3^, which is 1.8 and 1.4 times higher than those of the ambient. For samples collected in tunnel, platform and concourse, the differences in oxidative potential due to particle size and the oxidation potential due to PM mass concentration were not significant (p > 0.05).

### 3.2. Chemical compositions and characteristic metals

**Fig 3:**
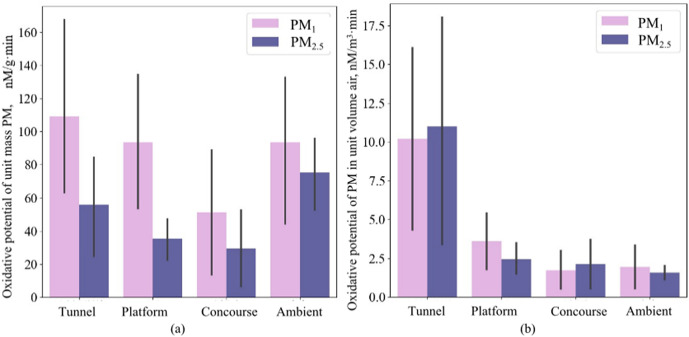
Oxidative potential of PM. (a) per unit mass PM. **(b)** PM per unit volume air.

**Fig 4:**
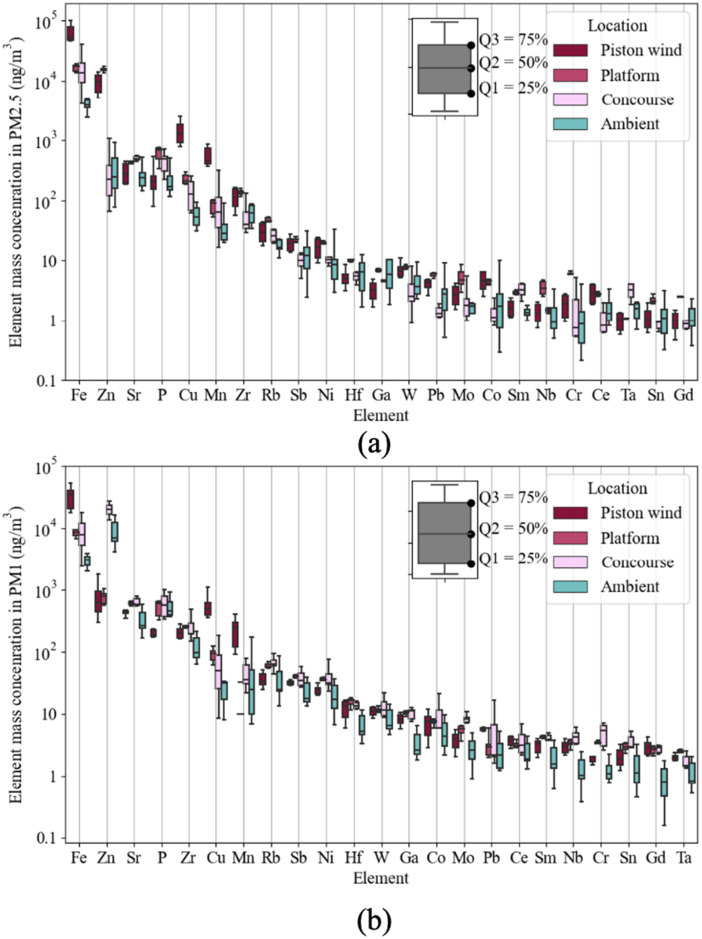
Element concentration in PM. **(a)** PM_2.5_ **(b)** PM_1_.

Concentrations of element and their ratio against ambient element concentration were demonstrated in Figs 3–4. Indoor Fe had an average element concentration of 16.3 μg/m^3^, the highest among tested elements. Metal concentrations in PM collected in the piston wind shaft were the highest among test locations. PM in the piston wind shaft was rich in Zn, Cu, Mn, and Fe, 10 times the ambient PM. Element on the platform concentrated on Zn and Cr compared with ambient, with their I/O ratio larger than 5. Metal elements concentrated on PM_2.5_, though the contrast has been demonstrated on the concourse. Metal brought to the concourse was PM_1_.

Compared with ambient PM (see Supplementary Material Section 3), Fe, Mo, Ni, and Mn, in the piston wind PM was characteristic. Mo, Ni was also found characteristic of the platform. Fe, Mn, and Cr were the main composition of hot-rolled steel rails for railways according to China standard GB/T 2585−2021 [46], with Mo, and Ni as trace elements. Therefore, it was inferred that the metal composition in PM was mainly from the train tunnel, due to the friction and mechanical process of the train wheel and railway.

Of all the detected elements, five elements in PM_2.5_ showed significant positive correlations with their oxidative potentials (Table 3). The main contributing elements to the oxidative potential of the DTT are the elements Fe, Mn, and Cu, with Fe and Mn having the largest coefficients and being the main contributors.

**Table 3:** Coefficients of correlation between elements and PM oxidative potential.

Element	Estimated Coefficient	Coefficient (CI = 95%)	p value
P	0.26	[0.02, 0.50]	p = 0.03
Co	0.28	[0.02, 0.53]	p = 0.03
Cu	0.63	[0.43, 0.83]	p < 0.001
Fe	0.70	[0.51, 0.88]	p < 0.001
Mn	0.69	[0.50, 0.87]	p < 0.001

### 3.3. Urinary biomarkers of inflammation and oxidative stress

The urinary biomarkers—MDA, IL-6, GSH, T-AOC, and 8-OHdG were compared between groups divided by background factors, including genders, smoking status, and BMIs (Supplementary Material Section 2). No significant difference was found between those groups, suggesting that the difference in biomarkers caused by gender, smoking, and BMI may be neglected.

**Fig 5:**
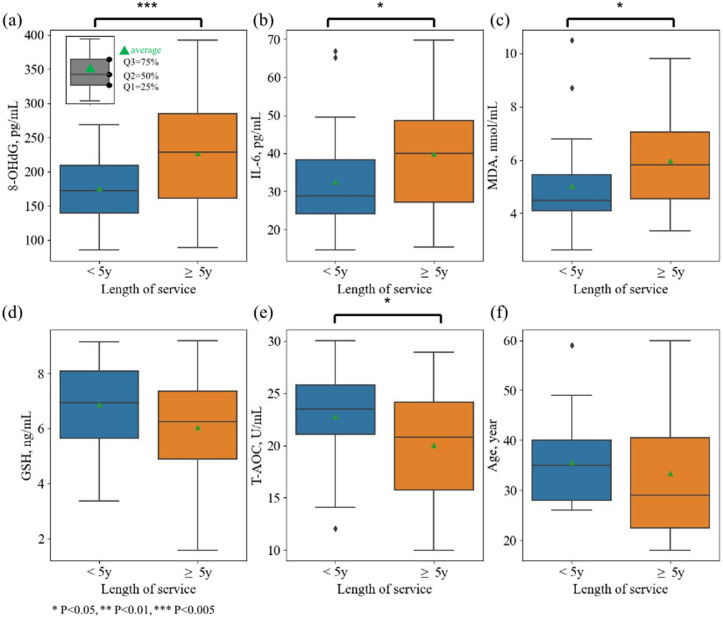
Comparison of urinary biomarkers between new (length of service < 5 years, N = 34) and senior employees (length of service ≥ 5 years, N = 40).

However, as shown in Fig 5, the urinary 8-OHdG, IL-6, MDA, and T-AOC among new employees (length of service < 5 years) varied significantly from senior employees (length of service ≥ 5 years). Average urinary 8-OHdG, IL-6, and MDA levels were significantly lower among senior employees. In contrast, average urinary GSH and T-AOC levels were lower, though the difference in GSH was not significant. The result proved that a higher oxidative stress level was demonstrated by 8-OHdG and MDA along with a lower antioxidant capacity, i.e., GSH, and T-AOC, among senior employees. The inflammation level shown by IL-6 was also higher for senior employees. However, the effect was not led by age, since there was no significant age difference found among new and senior employees. Linear mixed model showed similar result that only service of year had a significant relation with the biomarkers.

### 3.4. Change of biomarkers with accumulated exposure of PM and oxidative potential

Based on the group comparison of biomarkers, further correlation analysis was performed with the accumulated PM_1_, PM_2.5_ and element exposure. Linear mixed effect model showed no significant linear association of any biomarker with the accumulated PM_1_ exposure (p > 0.05). IL-6 had a significant linear correlation with the accumulated PM_2.5_ exposure (coefficient CI = 95%: [0.05,0.68], p = 0.025). With the increased accumulated PM_2.5_ exposure, the urinary IL-6 level of metro station staff presented an upward trend, inferring an increase in inflammation levels. No significant relation was found in other biomarkers after balancing background factors (results reported in Supplementary Material Section 3).

**Fig 6:**
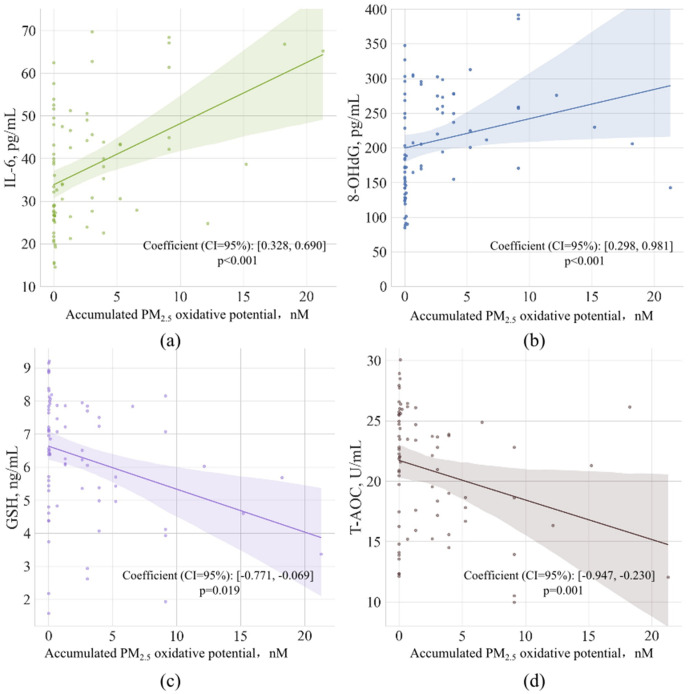
Significant linear association of biomarkers with accumulated oxidative stress from PM_2.5_ exposure. **(a)** IL-6, (b) 8-OHdG, **(c)** GSH, **(d)** T-AOC.

The accumulated oxidative potential of each volunteer had significant correlation with urinary biomarkers including IL-6, 8-OHdG, GSH and T-AOC as shown in Fig 6, coefficients were summarized in Table 4. This result suggested a significant increase of oxidative stress and a significant decrease of anti-oxidation capacity with the accumulative influence of oxidative potential rather than mass concentration for PM.

**Table 4:** Significant linear association of IL-6 and GSH with accumulated metal exposure of staff after balanced with length of service.

Biomarker	PM type	Element	Coefficient	CI = 95%	p value
IL-6	PM_2.5_	Ga	0.168	[0.01, 0.67]	0.05
		Mo	0.267	[0.12, 0.84]	0.01
		Ni	0.060	[0.02, 0.68]	0.04
		Sn	0.572	[0.02, 0.69]	0.04
	PM_1_	Co	0.157	[0.04, 0.73]	0.03
		Ga	0.121	[0.15, 0.86]	0.01
		Pb	0.480	[0.13, 0.85]	0.01
GSH	PM_2.5_	Mo	−0.017	[−0.71, −0.06]	0.02
	PM_1_	Co	−0.056	[−0.58, −0.01]	0.04
		Ga	−0.015	[−0.72, −0.07]	0.02
		Pb	−0.011	[−0.71, −0.06]	0.02

Metals in the PM contributed to the change of oxidative stress biomarkers. Linear mixed models were conducted for accumulated element exposure and biomarkers, balancing the length of service. Results with significant association were summarized in Table 4 (p < 0.05). Based on the statistical analysis, urinary IL-6 was significantly positive with several elements in PM, including Ga, Mo, Ni, Sn. A significant association was also found in GSH with a negative correlation with Mo. Similar to accumulated PM_2.5_ exposure, inflammation among metro stations may increase with the exposure of Ga, Mo, Ni, Sn accumulated. Meanwhile, antioxidant levels may be weakened.

## 4. Discussion

Blocked by full-height screen doors and controlled by mechanical ventilation, the PM_2.5_ concentration in the public area was lower than in previous studies under natural ventilation (driven by piston wind) [4] or in winter conditions when the ambient PM pollution was heavy [5,6]. Though PM_2.5_ may satisfy the standard for indoor air quality (GB/T 18883−2022) in China, it still failed to meet the requirement of WHO. Meanwhile, the hourly average I/O ratio of PM_2.5_ had an upward trend, demonstrating that the ventilation rate was insufficient to removal the indoor generated PM. Co and Mn are potentially hazardous metals in the fine particulate matter of the metro, and the concentration of Co in the station platform particulate matter has exceeded the acceptable risk of carcinogenicity (i.e., 10^−6^) recommended by U.S. Environment Protection Agency IRIS Health Risk Database [47]. Fe, Cu, and Mn were significantly higher in concentration and percentage than ambient PM_2.5_, and were characteristic of metro station PM, having significant correlations with the oxidative potential. Although P is significantly correlated with the oxidation potential results, the IRIS database does not provide recommended exposure limits for the above elements.

This study found that the urinary 8-OHdG, MDA, and IL-6 of new employees were significantly lower than senior employees, while the urinary T-AOC level was significantly lower. Urinary IL-6 and GSH levels of staff presented a statistically significant linear correlation with their accumulated Ga, Mo, Ni, Sn exposure levels. Background factors had little influence on the biomarker differences of volunteers. Previous work has compared the urinary 8-OHdG level among workers in the train tunnel and other places, with employees working in the tunnel showing a higher urinary 8-OHdG level than others [12]. Similar results were reported in the New York City metro system [13]. Though staff working in the tunnel was not recruited in this study, urinary 8-OHdG levels of senior workers were significantly higher than new workers, with no significant age difference found among groups. This result demonstrated the influence of accumulated exposure in the metro station public area may also induce oxidative DNA damage. The increase of oxidative stress was validated by other oxidative stress biomarkers including an increase in lipid peroxidation product MDA and a decrease in the total antioxidant capacity T-AOC.

According to the enrichment factors, Co, Mo, and Ni were characteristic elements found in the metro stations and were also found significantly correlated with the urinary inflammation and oxidative stress indicators. In this study, it was found that the combined influence led by particle size and chemical composition may be evaluated by oxidative potential using DTT assay rather than mass concentration. The result verified the statistically significant correlation between metro station staff’s PM oxidative potential exposure condition and their oxidative stress and inflammatory levels. Research from chronic exposure would help to speculate the health effect of metro station PM on commuters frequently using the metro system. Oxidative stress and inflammation were related to multiple diseases including immune system dysfunction [48,49], cardiovascular disease [50], and even neurodegeneration such as Alzheimer’s disease [51,52].

Metro is a significant part of world transport, delivering over 58 billion passengers annually. Thus, implementing stricter air quality monitoring and ventilation standards in metro systems could mitigate long-term public health risks. Given the high ridership and enclosed environments, proactive measures—such as real-time PM oxidation potential tracking and enhanced filtration—should be prioritized in urban transit policies to protect both staff and commuters.

The limitations of this study lay in the limited number of test metro stations and volunteers. Though the sample size was not enough to reach a firm epidemiological proof, this work aimed to conduct a pilot investigation of the PM pollution condition and its effect on metro station staff health. Another limitation was that the winter condition was not included. Though the elements mainly from the internal source may not change significantly, the ambient PM pollution would vary greatly in the heating season. Significant correlation between biomarkers and accumulated PM parameters were found among metro station staff, however, further research may be required to estimate the PM exposure for commuters due to the differences in exposure duration, age levels, etc.

## 5. Conclusion

This study investigated the PM pollution condition and its effect on metro station employees’ oxidative stress and inflammatory levels. Fe, Mo, Ni, and Mn were identified as characteristic metals of the metro station PM. Meanwhile, urinary 8-OHdG, MDA, and IL-6 of new employees were significantly higher than senior employees (length of service in the metro station > 5 years). Co, Mo, Ni, Sn, Ga and Pb were found significantly correlated with urinary IL-6 and GSH. However, the accumulated PM_2.5_ mass concentration was not significantly correlated with any biomarkers except IL-6. The result inferred that the impacts from metals cannot be ignored when estimating the health risk of metro station particle matters. The oxidative potential using DTT method is a potential estimation to integrate the combined influence led by particle size and chemical composition. Co, Mo, and Ni were characteristic elements found in the metro stations and were also found significantly correlated with the urinary inflammation and oxidative stress indicators. The accumulated oxidative potential exposure of PM_2.5_ mass concentration was significantly correlated with IL-6, 8-OHdG, GSH, and T-AOC after balancing background factors. Results indicated that chronic metal PM exposure in the metro station may induce oxidative stress and inflammation, suggesting oxidative potential assessed by DTT would be a better health effect evaluation method for PM control than mass concentration.

## Supporting information

S1 File Supplementary material for *Increased inflammation and oxidative stress caused by accumulated metal particle exposure among metro station staff.* (DOCX)
