# Supplementary material for: Increased inflammation and oxidative stress caused by accumulated metal particle exposure among metro station staff
Source: PLoS One. 2025 Dec 10;20(12):e0337592. doi: 10.1371/journal.pone.0337592 (PMC12694840; doi:10.1371/journal.pone.0337592)
Supplement: S1 File — (DOCX) [file pone.0337592.s001.docx]

Supplementary material for

*Increased inflammation and oxidative stress caused by accumulated metal particle exposure among metro station staff*

*Zukun Wang^1^, Junjie Liu^1^, Yijun Song, Mingyao Yao^1^, Shihao Wen^1^, Wenzhe Shang^1^, Mingtong He^1^, Yushuang Wang^1^, Junjie He^1^*

^1^Tianjin Key Laboratory of Indoor Air Environmental Quality Control, School of Environmental Science and Engineering, Tianjin University, Tianjin, China

^*^Corresponding author.

Junjie Liu, Email: [jjliu@tju.edu.cn](mailto:jjliu@tju.edu.cn)

Yijun Song, Email: songyijun@ihcams.ac.cn

# Oxidative potential analysis

The dithiothreitol (DTT) method was applied as a cell-free method for analyzing the oxidative potential (OP) of PM following steps described in previous studies [6, 43]. In brief, a quarter of a quartz filter was cut into pieces using ceramic scissors and then extracted with 15 mL of deionized water. 150 μL of 10 mM DTT was added into the mixture, incubated at 37 °C in a water bath for 30 min after well mixed. Then, 1.5 mL of the incubation mixture was extracted with 1.5 mL of 10% trichloroacetic acid (Aladdin Reagent Co., Ltd, Shanghai, China) added to stop the reaction. 250 μL of 0.01 M DTNB (5,5'-Dithiobis-(2-nitrobenzoic acid)) was added and allowed to react for 2 min. 6 mL of 0.40 M Tris-HCl (Tris Hydrochloride) buffer with a pH 8.9 was added in the mixture with 20 mM EDTA (Ethylenediaminetetraacetic acid) to ensure that the sample remained quenched until the DTT reacted with the DTNB. The DTT, DTNB, EDTA, and Tris-HCl buffer were obtained from Shanghai Yuanye Bio-Technology Co., Ltd, Shanghai, China. The total volume allowed repetitive measurements within 30 min of the absorbance (Abs) for the reaction product (2-nitro-5-thiobenzoic acid, TNB) at 412 nm with a spectrometer (TU-1810, Persee Scientific Instrument Co., Ltd. Beijing, China). Tests of the blank quartz filters and samples were all run in triplicate.

The OPs of the PM samples were reported as the calculated DTT consumption rate. A standard curve was prepared with different DTT concentrations (0, 0.08 mM and 0.1 mM) to calculate the amount of DTT consumed during the reaction. The consumption rate was linear when less than 20% of the DTT was depleted (R > 0.99). The DTT consumption rate was calculated as follows:

$$\begin{aligned} Abs=k\cdot C+b\#(3) \end{aligned}$$

$$\begin{aligned} C_{sample}=C_{0}-\frac{Abs_{sample}-b}{k}+C_{blank}\#\left( 4 \right) \end{aligned}$$

$$\begin{aligned} OP=\frac{C_{sample}\cdot V}{T\cdot m_{sample}}\cdot1000\#\left( 5 \right) \end{aligned}$$

In equation (4), $C_{sample}$ is the DTT concentration consumed by the PM sample during the reaction, mM; $C_{0}$ is the initial DTT concentration, 0.1 mM in this work; $Abs_{sample}$ is the TNB absorbance of the PM sample and $C_{blank}$ is the average DTT consumption of the blanks, which is neglectable in this work, so, $C_{blank}\approx0$. In equation (5), $OP$ is the DTT activity of the PM, the concentration consumption rate normalized by the PM mass ($m_{sample}$, mg) involved in the reaction, μmol/(mg·min); $V$ is the volume of DTT used in the assay, mL; and $T$ is the reaction time, min.

# Comparison of urinary biomarkers by background factors


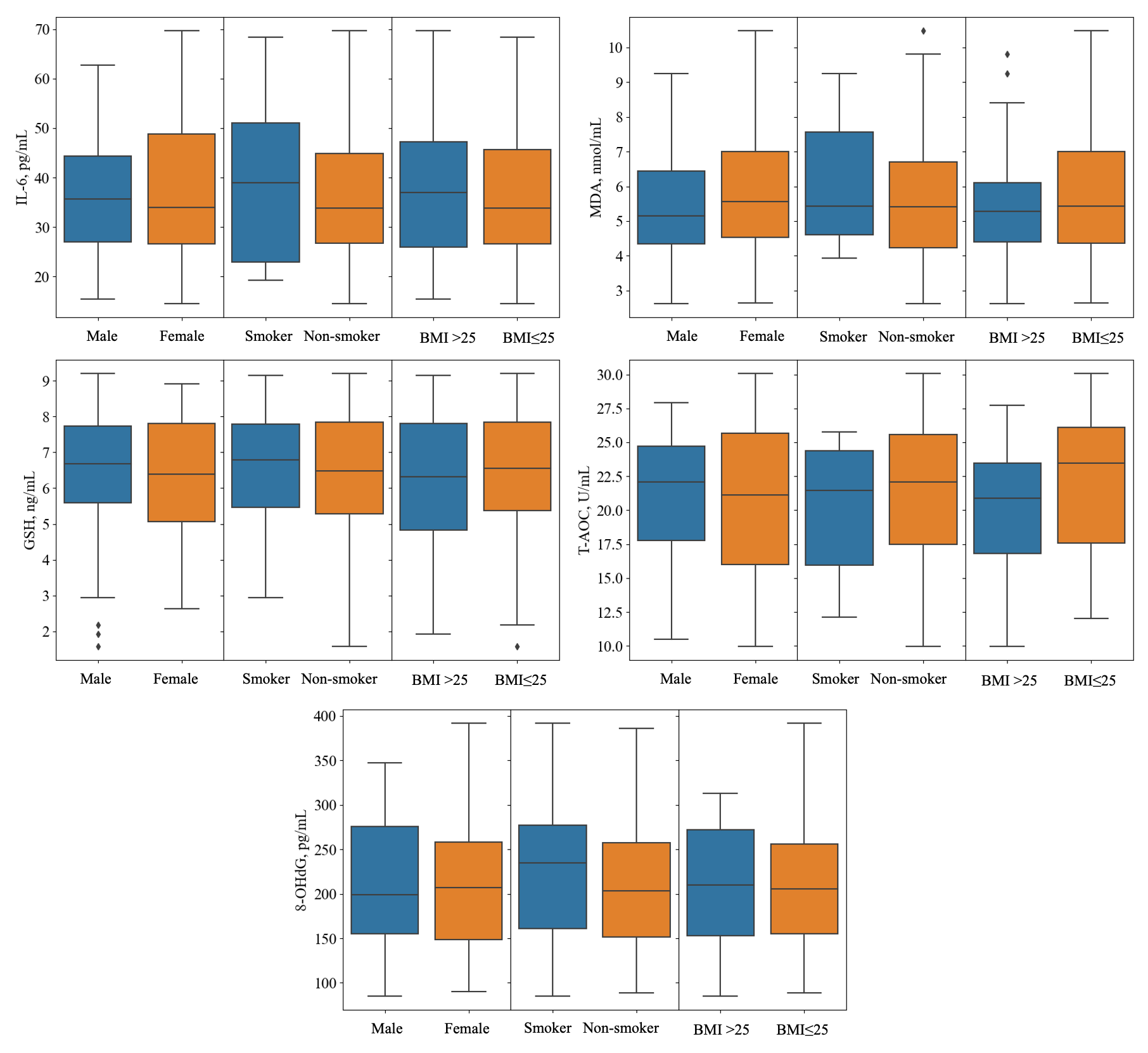


Figure 1 Comparison of urinary biomarkers between gender, smoker and non-smoker, obesity and non-obesity group.

# Chemical Compositions and Characteristic Metals

| 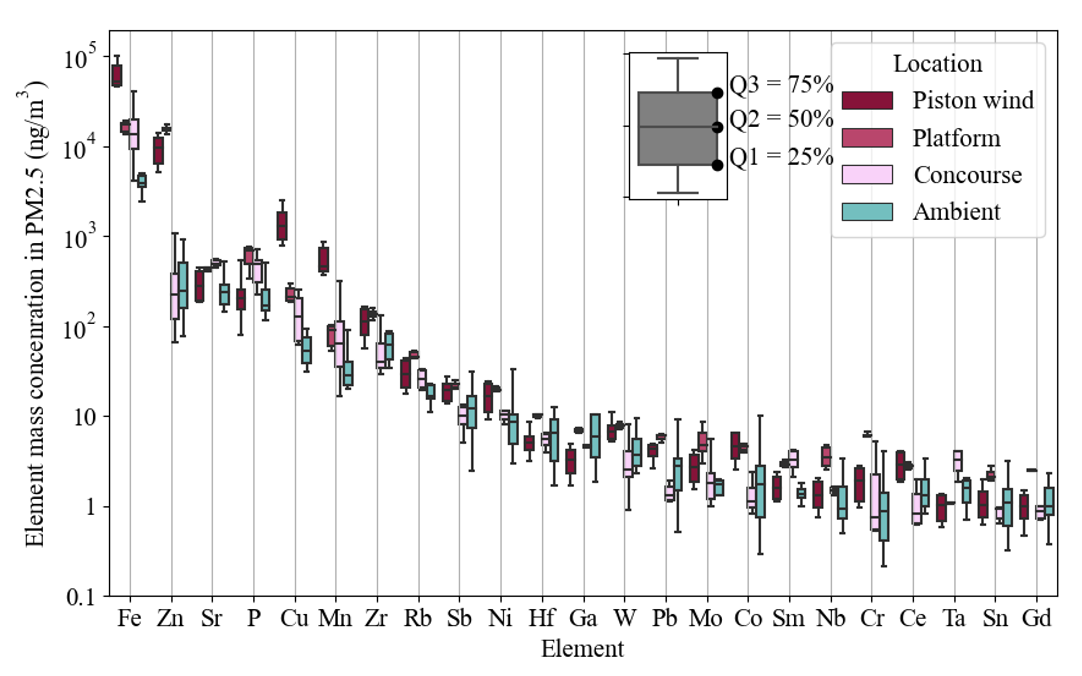  (a) |
| --- |
| 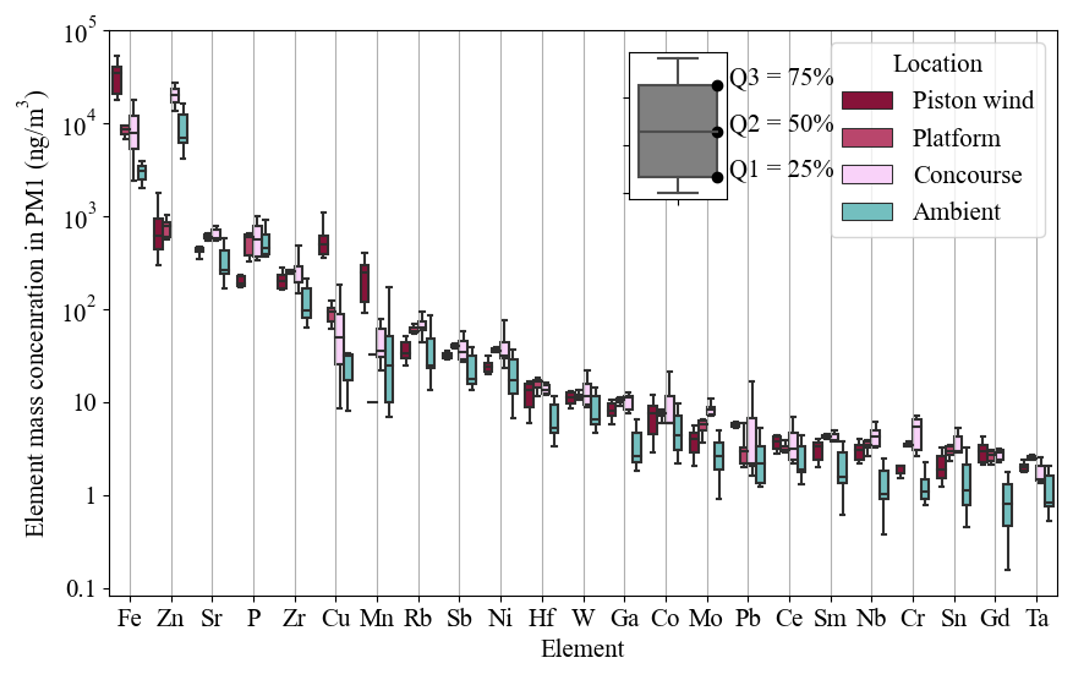  (b) |

Figure 2 Element concentration at different locations in PM. (a) PM_2.5_ (b) PM_1_

| 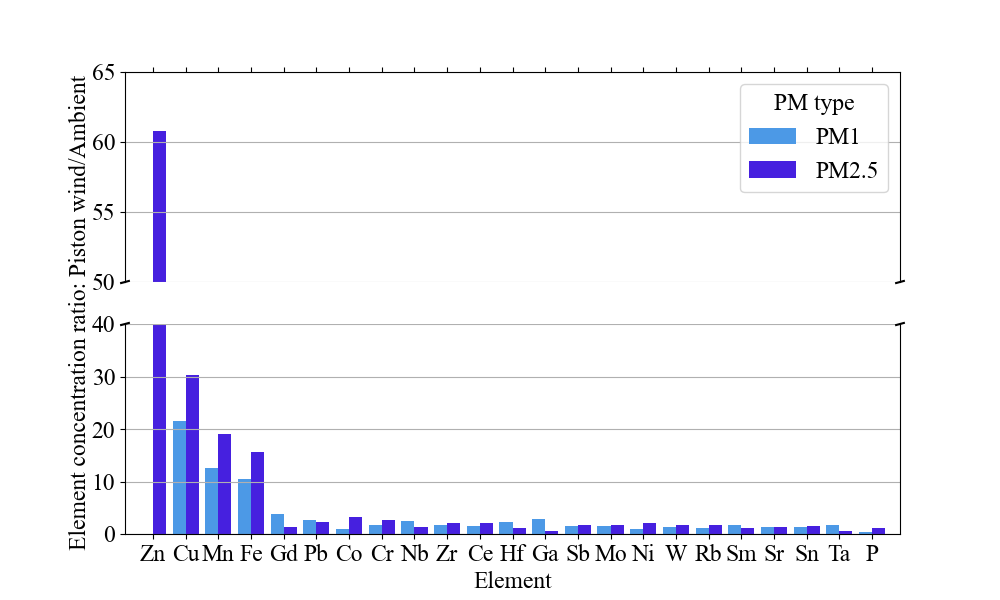  (a) |
| --- |
| 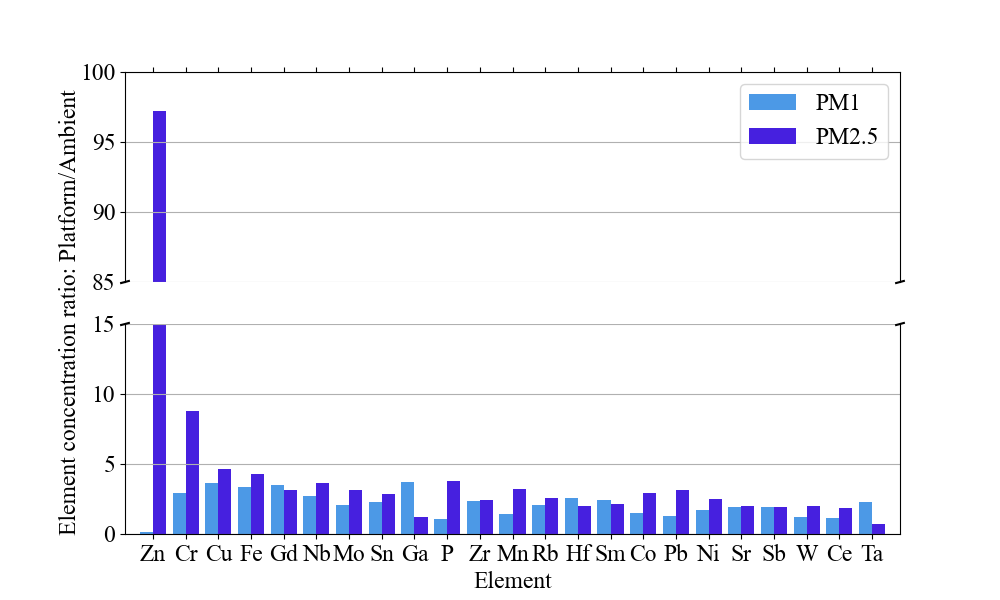  (b) |
| 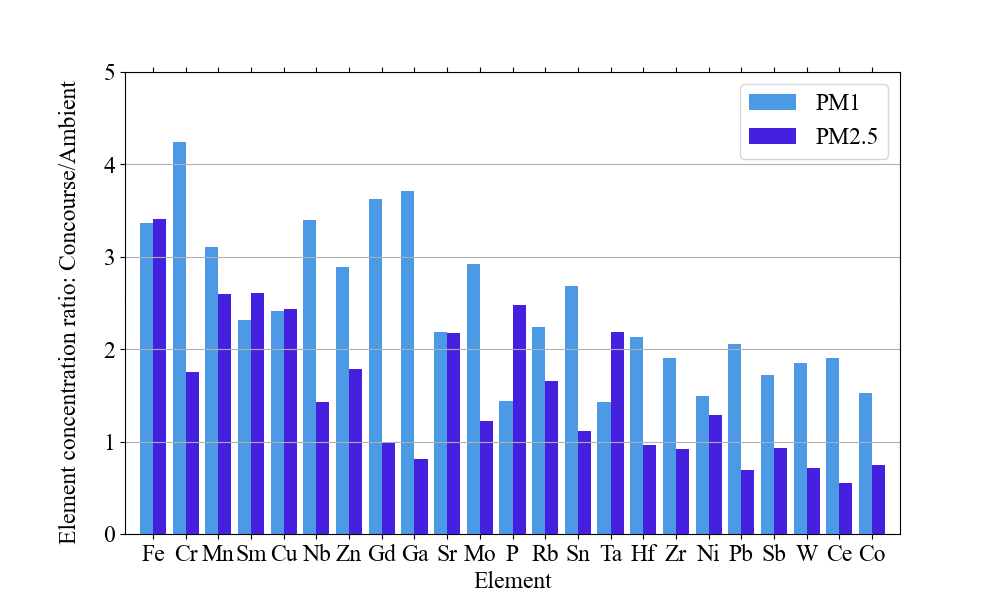  (c) |

Figure 3 Average indoor/outdoor ratio at different locations

(a) piston wind shaft, (b) platform, (c) concourse

| 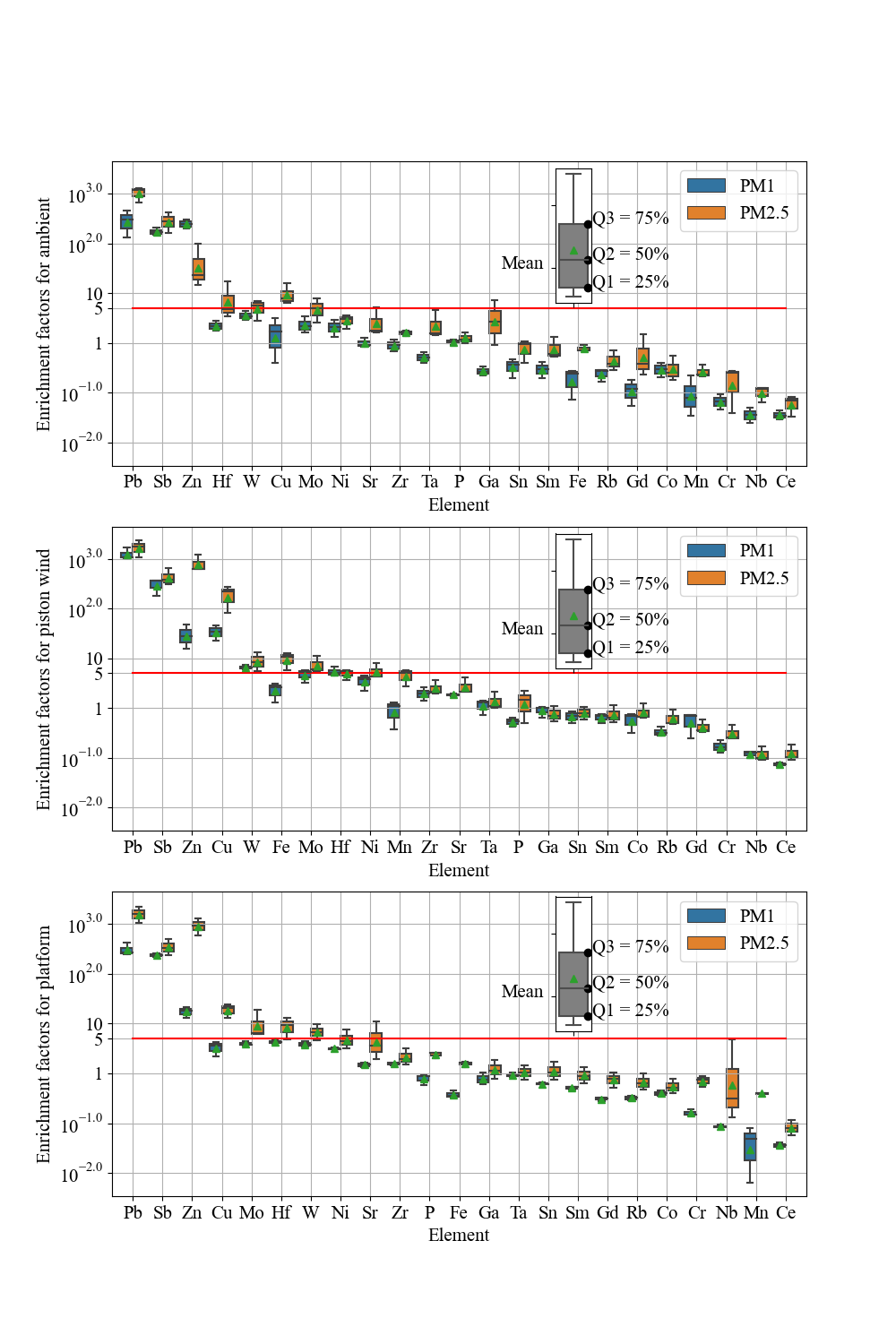  (a) |
| --- |
| 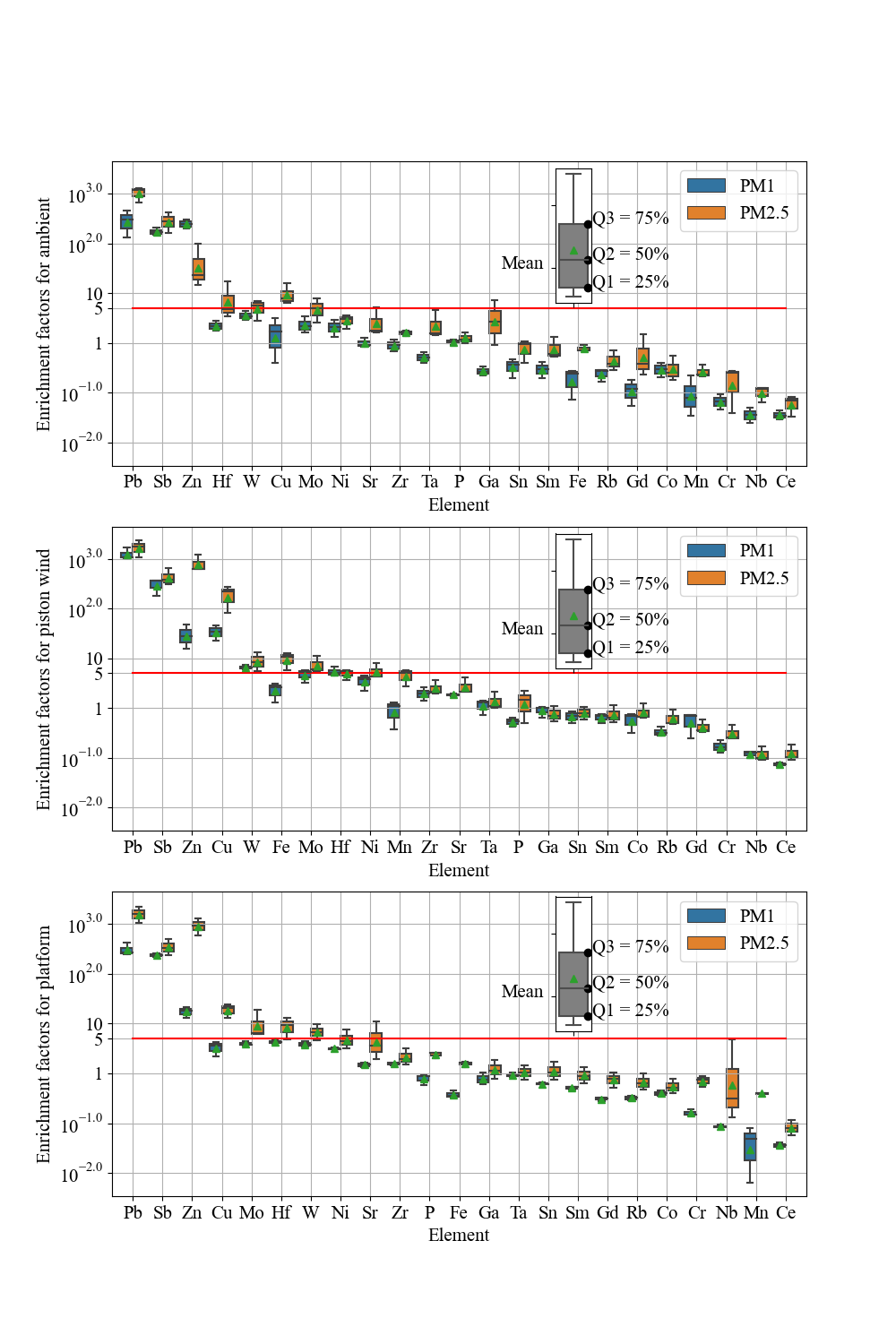  (b) |
| 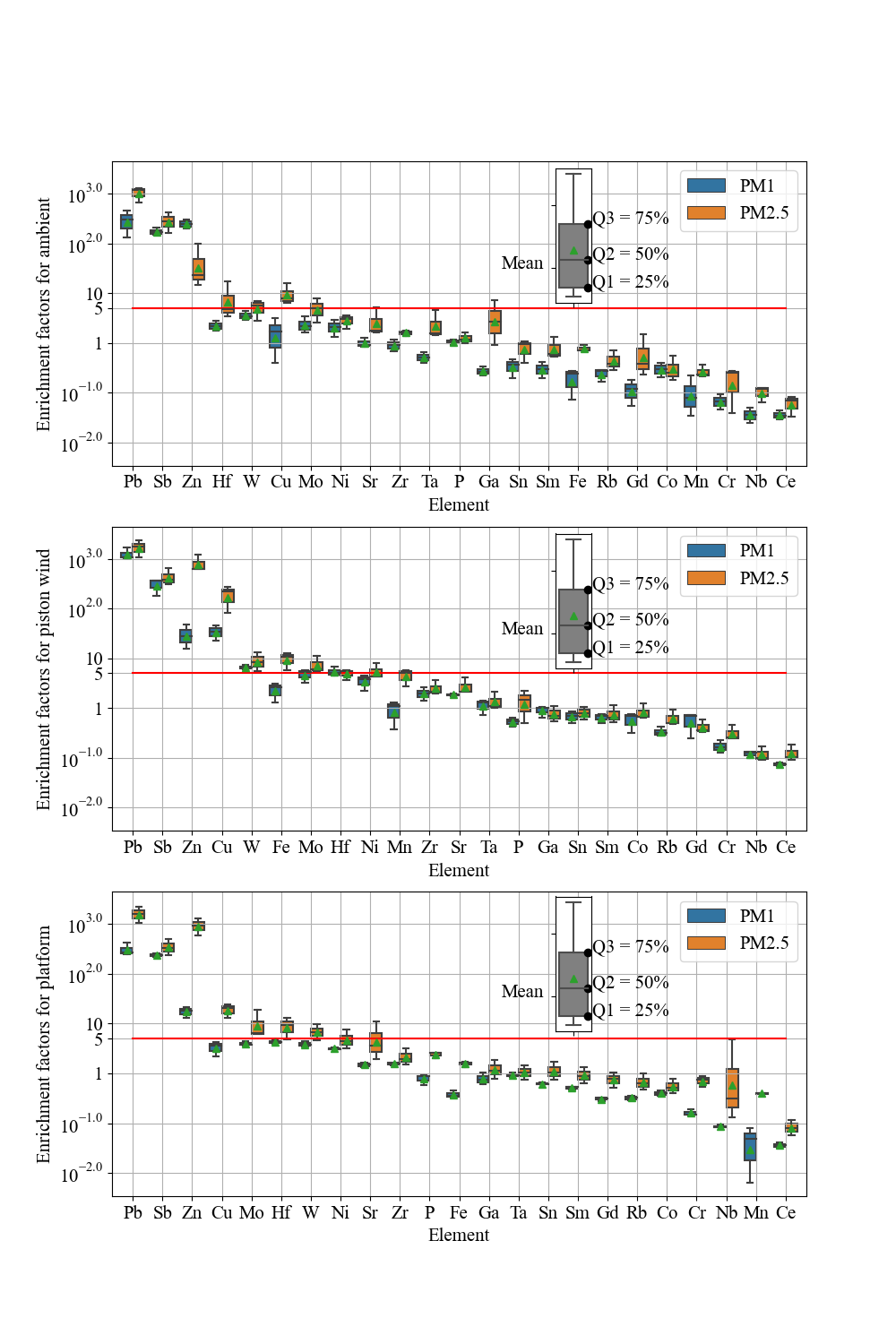  (c) |

Figure 4 Average enrichment factors of PM in different places

(a) ambient, (b) piston wind shaft, (c) platform

# Urinary biomarkers change along with accumulated PM exposure


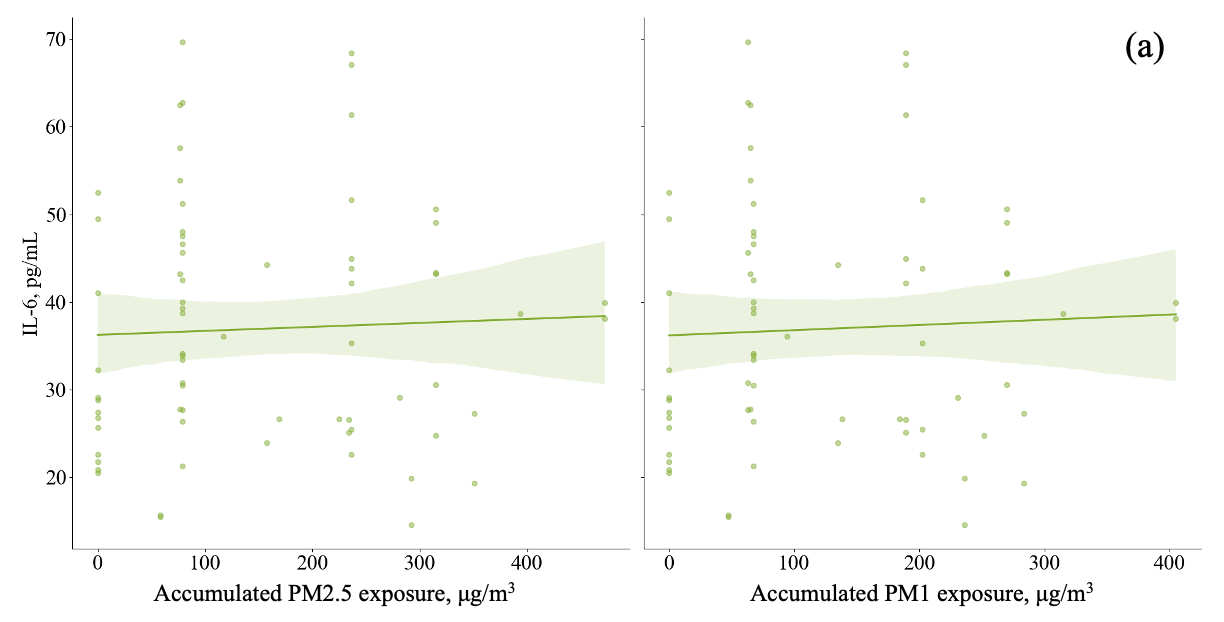

Figure 5 Urinary biomarkers and accumulated PM exposure of each staff

(a) IL-6, (b) MDA, (c) GSH, (d) T-AOC, (e) 8-OHdG

# Data DOI

The data set required was uploaded to https://edirepository.org/ and could be fetched though the following doi: [https://doi.org/10.6073/pasta/c1d051556fd2d387b7c6c0e3f2e3cda8](https://doi.org/10.6073/pasta/c1d051556fd2d387b7c6c0e3f2e3cda8).
